# Malignant Transformation of Unknown Duration of an Ovarian Mature Cystic Teratoma Presenting as a Trocar Recurrence in a Young Patient: A Case Report and Literature Review

**DOI:** 10.1155/2023/8875092

**Published:** 2023-11-21

**Authors:** Tomohiro Okuda, Yoko Uda, Shiho Sakai, Taishi Harada

**Affiliations:** ^1^Department of Obstetrics and Gynecology, Fukuchiyama City Hospital, Fukuchiyama City, Kyoto, Japan; ^2^Department of Medical Oncology, Fukuchiyama City Hospital, Fukuchiyama City, Kyoto, Japan

## Abstract

Although laparoscopic cystectomy is a safe and effective management strategy for ovarian mature cystic teratoma (MCT) in pediatric and adolescent patients, it has been challenged because of its association with a higher risk of intraoperative spillage leading to chemical peritonitis, adhesion formation, and iatrogenic implantation of malignant cells. Here, we report a rare case of a 23-year-old female patient with MCT tissue during laparoscopic ovarian cystectomy that remained in the peritoneum, possibly becoming malignant thereafter. Intraoperatively, the cyst's contents leaked into the abdominal cavity. The abdominal cavity was thoroughly cleaned before the operation was completed. Pathological examination revealed an MCT without malignant findings. The patient's postoperative course was uneventful. Although the excised tissue was benign, the patient presented with a mass at the trocar wound (upper suprapubic area) 2 years after initial surgery. Biopsy results indicated squamous cell carcinoma. Moreover, peritoneal and bladder invasions were diagnosed. She subsequently experienced symptoms of cancerous peritonitis. Achieving a complete cure through surgery alone was deemed difficult; however, successful neoadjuvant chemotherapy and tumor reduction surgery kept her alive up until the publication of this case report, 3 years since diagnosis with squamous cell carcinoma. This case indicates that malignant transformation of MCTs can occur at any age.

## 1. Introduction

Laparoscopic cystectomy is a safe and effective method for managing ovarian mature cystic teratomas (MCTs) in children and adolescents [1], while malignant transformation is a rare complication of ovarian MCTs [2]. Herein, we describe the case of a 23-year-old female patient who was diagnosed with squamous cell carcinoma (SCC) caused by malignant transformation of tissue adherent to trocar holes or trocar recurrence 2 years after undergoing laparoscopic ovarian cystectomy for a dermoid cyst. Although the excised tissue was benign, SCC was identified at the trocar foramen 2 years later. The clinical course suggests that the MCT tissue attached to the peritoneum had become malignant.

## 2. Case Presentation

**Figure 1:**
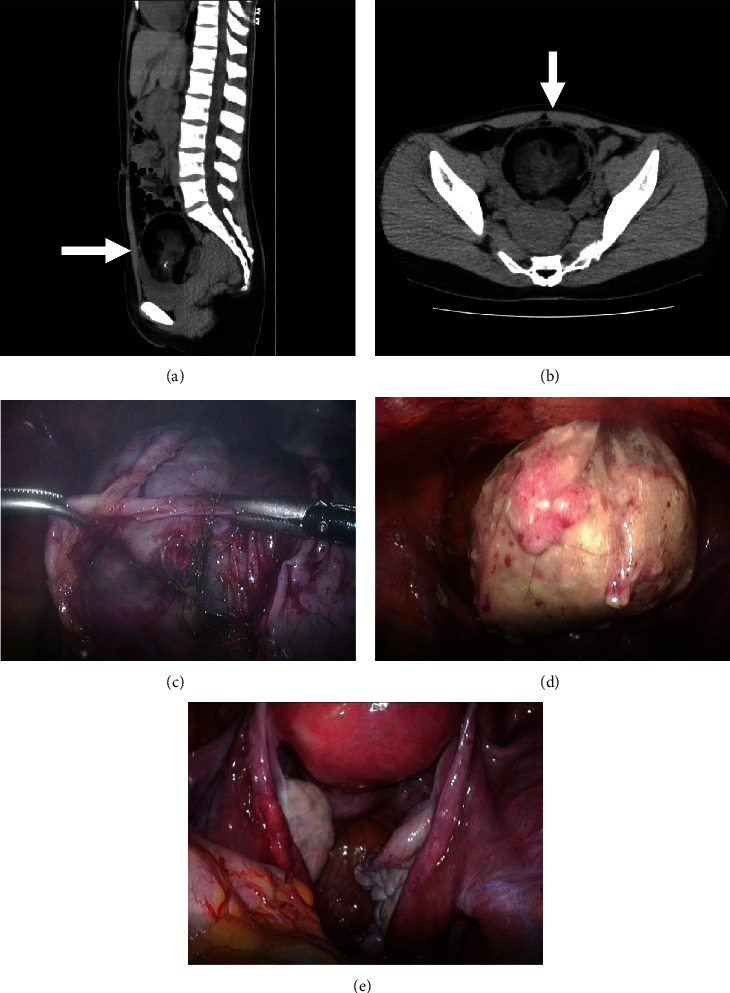
Pelvic computed tomography showing a dermoid cyst. No malignant findings (a, b, white arrow) are noted. Intraoperatively (c), the cyst was removed from the body using a bag (d), and the pelvic cavity was cleaned (e).

A 23-year-old female patient with no history of pregnancy suddenly experienced severe lower abdominal pain before going to bed, which intensified and became unbearable; she therefore visited our hospital late at night on a national holiday. A dermoid cyst was suspected following computed tomography (CT). However, the CT scan did not reveal any imaging findings indicative of malignancy (Figures 1(a) and 1(b)). Because of the sudden onset of the disease, torsion or rupture of an ovarian cyst was suspected. Magnetic resonance imaging (MRI) could not be performed because of the unavailability of emergency MRI services. Eventually, emergency surgery was performed because analgesics were ineffective. Because of her young age, the patient underwent laparoscopic surgery via a 5 mm trocar in the umbilicus, right and left lower midsections, and a 12 mm trocar in the suprapubic region. Laparoscopic ovarian cystectomy was performed for a ruptured cyst (Figure 1(c)). During cystectomy, care was taken to prevent spillage of the tumor contents (mainly fat and hair), and the excised tissue was placed in a bag and removed via the upper pubic trocar hole (Figure 1(d)). The abdominal cavity was thoroughly cleaned (Figure 1(e)), and the patient was discharged on postoperative day 3. Histopathological examination revealed an MCT with no malignant findings.

**Figure 2:**
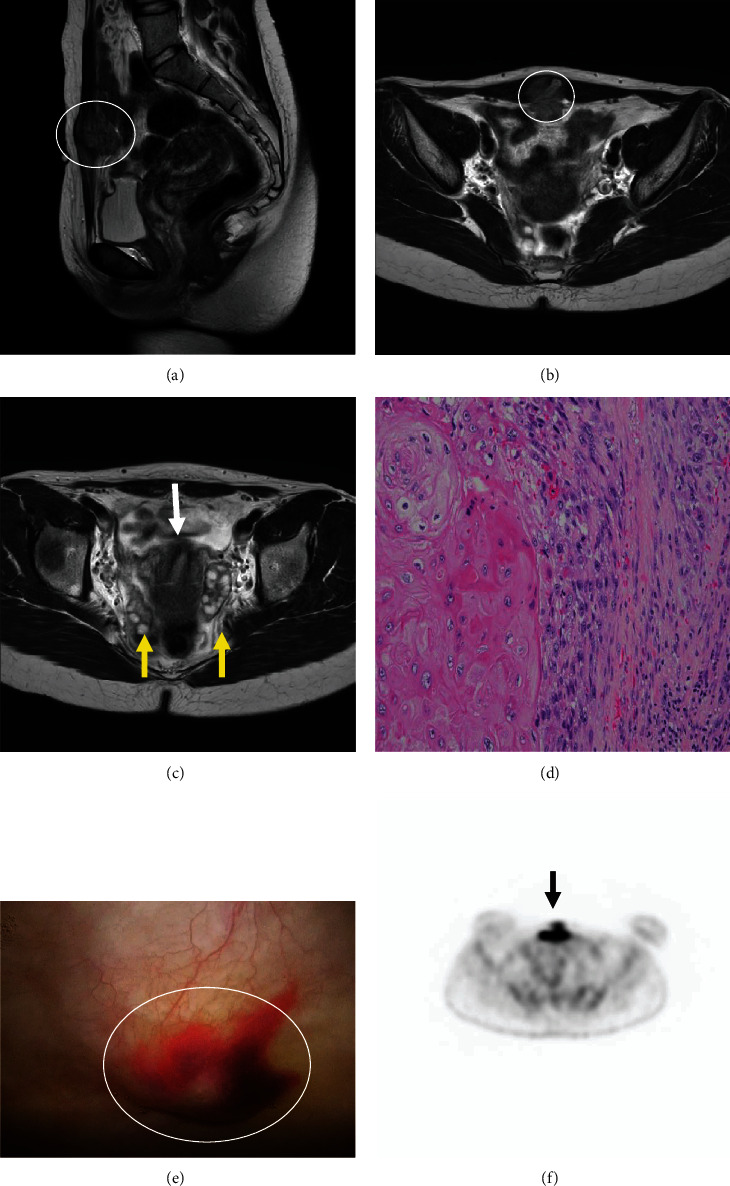
Magnetic resonance imaging showing a subcutaneous mass with an irregular surface (a, sagittal view, white circle; b, horizontal view, white circle) and with a normal uterus (c, white arrow) and ovaries (c, yellow arrows). Biopsy results reveal an invasive squamous cell carcinoma with carcinomatous nodule formation (d). Cystoscopy reveals cancerous invasion of the bladder (e, white circle). Fluorodeoxyglucose accumulation was observed in the lower abdominal wall (f, black arrows).

Two years after the initial surgery, the patient visited the hospital because of abdominal pain around a mass in the trocar wound (upper suprapubic area). The mass had irregular margins and was palpable. MRI revealed an indistinct mass in the 3 cm range, suspected of being either a primary tumor of the abdominal wall, carcinoma originating from a ureteric duct remnant, or malignant transformation of a teratoma attached to the peritoneum (Figures 2(a) and 2(b)). No utero-ovarian abnormality was noted (Figure 2(c)). Biopsy revealed invasive SCC with carcinomatous nodule formation (Figure 2(d)), and the SCC-related antigen level increased to 5.1 ng/mL (2 years prior, SCC-related antigen level was 1.4 ng/mL (normal range < 1.5 ng/mL)). Therefore, tissue from the site of the initial surgery was re-excised, and additional pathological tests were performed. However, histopathological examination of the excised tissue revealed no evidence of malignancy.

Although carcinomas may arise from remnants of the ureteric duct, they are generally located at the bladder apex, and their histologic findings are similar to adenocarcinoma [3]. Moreover, SCC of cutaneous origin was deemed unlikely in this case because of the deep location of the primary tumor site. Therefore, we concluded that either the residual tissue of the MCT attached to the surgical wound of the suprapubic trocar puncture site had undergone malignant transformation or a malignant tumor was present during the initial surgery and a port-site metastasis had developed. We believed that malignant transformation had already begun during the initial surgery; however, because of its extremely small size, it could not have been diagnosed by the excised pathology specimen and may have subsequently developed as a trocar recurrence.

Additional tests were performed to determine if radical resection of the abdominal wall around the trocar hole was feasible. Although MRI showed no utero-ovarian abnormalities, cystoscopy revealed cancerous invasion of the bladder (Figure 2(e)). Positron emission tomography-CT (PET-CT) was performed to confirm the presence of metastases. Fluorodeoxyglucose accumulation (maximum standardized uptake value = 6.93) was observed in the lower abdominal wall (Figure 2(f)). Therefore, it was determined that cure would be difficult using surgery alone.

Subsequently, the patient experienced symptoms of cancerous peritonitis and decided not to undergo radical surgery. After discussion with a multidisciplinary team of doctors, we decided to treat the patient with a first-line chemotherapy regimen comprising nab-paclitaxel (100 mg/m^2^) plus carboplatin (CBDCA), with an area under the curve of 6, plus pembrolizumab chemotherapy (200 mg) and pembrolizumab maintenance chemotherapy. After four cycles of chemotherapy, the patient showed a partial response and was treated with pembrolizumab maintenance therapy; however, the abdominal pain worsened, and CT scan revealed that the tumor had increased in size. The treatment strategy was revised, and radiation therapy was initiated with a second-line palliative chemotherapy regimen comprising cisplatin (cis-diamminedichloroplatinum (II), CDDP) of 80 mg/m^2^+S-1 (tegafur, gimestat, and otastat potassium) of 80 mg/m^2^ (SP3)+50 Gy radiation (2.0 Gy/day, days 1–5/w × 5 total: 50 Gy). Unfortunately, due to intense bone marrow suppression, only one dose of CDDP was completed, and S-1 was continued as maintenance therapy.

Fortunately, chemoradiation therapy was successful, and a complete response was observed on imaging. Six months after chemotherapy was initiated, PET-CT revealed a suspected recurrent lesion on the right side of the uterus in the irradiated area, and the patient underwent reduction surgery. Total hysterectomy, bilateral salpingo-oophorectomy, omentectomy, peritoneal dissection, and abdominal wall resection/plasty were performed. The abdominal wall could be closed as usual without requiring skin valvuloplasty. Pathology results revealed that residual invasive SCC was only detected on the right fallopian tube angle. Complete resection was achieved through surgery; however, the patient requested adjuvant chemotherapy. Therefore, CBDCA/pegylated liposomal doxorubicin (PLD) chemotherapy, comprising PLD (50 mg/m^2^) plus CBDCA, with an area under the curve of 6, was administered over four cycles. Finally, the patient was alive for 3 years with complete response after the initiation of chemotherapy. The patient provided informed consent for publication of the case data.

## 3. Discussion

Benign ovarian cysts in women of reproductive age are common; indeed, by the age of 65 years, 1 in 25 females is admitted to the hospital for an ovarian cyst [4]. MCTs are the most common histological type of benign ovarian tumors among women of reproductive age, accounting for 40–50% of all benign ovarian neoplasms [5]. MCTs are germ cell tumors of the ovaries originating from the ectoderm, mesoderm, and endoderm and are termed dermoid cysts if the ectodermal tissue predominates other germ cell layers [6]. Laparoscopic cystectomy is a safe and effective method for managing ovarian dermoid cysts in pediatric and adolescent patients [1]. However, laparoscopic ovarian cystectomies for MCTs are associated with two limitations: intraoperative rupture and malignant transformation. Nezhat et al. previously reported that laparoscopic surgery has an intraoperative rupture rate of 15–100%, whereas open surgery has a lower intraoperative rupture rate of 4–13% [6], which is a more acceptable figure. Childress et al. previously reported that although laparoscopic cystectomy of ovarian dermoid cysts is linked to a greater likelihood of intraoperative cyst rupture, minimally invasive surgery for ovarian dermoid cysts is a reasonable approach because cyst rupture is rarely associated with complications [7]. Our patient had an uneventful postoperative course and was discharged on postoperative day 3 without major complaints for a reasonable duration.

Malignant transformation of ovarian MCTs is a rare phenomenon that usually occurs in postmenopausal women [8]. SCC originating from the cyst's squamous lining is the most common type of malignant transformation, accounting for 80–83% of cases, followed by adenocarcinoma (7%) and sarcoma (7%) [8]. Rha et al. reported that ovarian teratomas that become malignant appear as fat-containing tumors with enhanced, irregularly margined solid components on CT and MRI scans [9]. Even without evidence revealing malignancy, such as a solid component on imaging, an elevated SCC-related antigen level may indicate malignant transformation. Specifically, Chiang et al. showed that postmenopausal age, large tumor size (≥15.0 cm), and solid components in MCTs are indicators of malignant transformation risk [10]. Our patient was young, the SCC-related antigen level was within the normal reference values during the first operation, the tumor diameter was 10 cm, and the characteristics of fat-containing tumors with enhanced, irregularly margined solid components on CT (as reported by Rha et al.) were not observed. Moreover, no evidence of malignancy was found in the excised tissue. We therefore concluded that the invasive SCC in this case likely originated from the MCT tissue attached to the abdominal wall during the original laparoscopic surgery which subsequently recurred and became malignant.

Generally, dermoid cysts > 8 cm in patients aged <30 years are prone to recurrence [11]. Moreover, the incidence of recurrent dermoid cysts in pediatric and adolescent populations following ovarian cystectomy is 10.6% [12]. If the contents of a dermoid cyst are predominantly serous, ovarian cyst enucleation following puncture aspiration could reduce leakage. However, if the cyst has a high fatty component, the contents may leak into the abdominal cavity even if puncture aspiration is attempted. Moreover, even if a bag is used, the contents can adhere to the outside of the bag. In our case, although a bag was used to remove the tissue from the body, tumor contents were still found outside the bag. Importantly, we cannot rule out the possibility that a relatively small amount of malignant tissue, undetected by tumor markers, was present during the initial surgery.

The patient's SCC-related antigen level was within the normal threshold during the first procedure, and the excised tissue showed no evidence of malignancy. However, the SCC-related antigen level was elevated when the patient was re-examined after noting an abdominal wall mass. Although it is a matter of speculation, the tissue left behind may have undergone malignant transformation.

In conclusion, although port-site metastasis could not be ruled out, we encountered what we believe to be an extremely rare case of malignant transformation of an ovarian MCT where tissue adherent to the abdominal wall trocar foramen underwent malignant transformation in a young patient. However, consensus on the ideal treatment is lacking because of its rarity. This case indicated that malignant transformation of MCTs can occur at any age. Because the preoperative diagnosis of malignant transformation of dermoid cysts in young women is difficult without abnormal imaging findings or tumor markers, other MCT cases may show distinct characteristics; therefore, management should be individualized.

## Data Availability

Data sharing is not applicable to this article, as no datasets were generated or analyzed during the current study.
